# Practical tools for female-specific ADHD: The impact of hormonal fluctuations in clinical practice and from the literature

**DOI:** 10.1192/j.eurpsy.2025.10120

**Published:** 2025-10-21

**Authors:** Dora Wynchank, Maxime de Jong, Sandra J. J. S. Kooij

**Affiliations:** 1 PsyQ, Expertise Centre Adult ADHD, The Hague, The Netherlands; 2 Department of Psychiatry, Amsterdam UMC/VUmc, Amsterdam, The Netherlands; 3 Amsterdam Public Health Research Institute, VU Medical Centre, Amsterdam, The Netherlands

**Keywords:** attention deficit/hyperactivity disorder, female, female-specific therapy, premenstrual, sex hormones

## Abstract

Hormonal fluctuations significantly impact women with attention deficit/hyperactivity disorder (ADHD), affecting symptom severity, mood, sleep, and treatment efficacy. Many women report cyclical variations in symptom intensity and reduced psychostimulant efficacy during the late luteal phase of their menstrual cycle. Also, during the postpartum period and in the (peri)menopause, ADHD symptoms may worsen, accompanied by increased mood and sleep disturbances. Neglecting these features specific to women with ADHD has resulted in underdiagnosis and misdiagnosis of ADHD, as well as suboptimal treatment. In addition, the accuracy of ADHD diagnosis in women is complicated by symptom masking, comorbid anxiety/depression, and referral biases. To help improve care for women with ADHD, we provide practical recommendations for assessing the impact of hormonal fluctuations in ADHD research and practice (e.g., a protocol for assessment, including menstrual cycle tracking and validated questionnaires for mood and sleep disturbances). Our recommendations are informed by extensive clinical experience and research initiatives focused on women with ADHD. We briefly describe the specifics of clinical presentation, premenstrual exacerbations of these women, and their optimal treatment. We also suggest tailored assessment, such as considering hormonal status in ADHD diagnosis and symptom tracking across menstrual, postpartum, and (peri)menopausal phases. While more research is sorely needed, recognising and identifying these hormone-related fluctuations is crucial for improving research practices and clinical management of women with ADHD.

## Introduction

Historically, attention deficit/hyperactivity disorder (ADHD) in girls and women remains under- or misdiagnosed leading to significant personal and societal consequences [1]. Girls under 12 years are diagnosed with ADHD almost four times less often than boys (1:4.8), but this ratio becomes closer to equal by adulthood [2, 3]. This suggests that many girls are overlooked during childhood, leading to delayed diagnoses [1]. A lack of awareness among clinicians may underlie this delay in diagnosis [4], while other factors such as differences in symptom presentation [5]; “male-stereotype” diagnostic criteria [6]; parental, referral, and informant bias may also influence diagnosis in girls [5, 7]. Additionally, hormonal fluctuations significantly impact women with ADHD. Therefore, sex and gender differences, as well as psychosocial expectations must be considered to improve female-specific research and clinical practice.

## ADHD and female gender: Psychosocial expectations and contextual burden

Currently, girls may not meet diagnostic thresholds unless their symptoms are particularly severe or accompanied by additional problems, such as emotional difficulties or academic impairment [8]. Masking of symptoms in girls and comorbid anxiety and depression also complicate the diagnosis of ADHD; these factors may result in a low index of clinical suspicion for ADHD in females [1, 8, 9]. Furthermore, developmental, social, and cultural influences can affect the accuracy of diagnoses throughout the female lifespan. For instance, societal norms play a significant role in shaping perceptions of appropriate behaviour, and these norms may vary by gender [4] and ethnic group [10, 11], potentially impacting how symptoms are recognised and interpreted. The novelty of research on female ADHD may also play a role [12]. (Recent) research describes distinct cognitive [13], social [14], clinical [15], psychiatric [16, 17], neurochemical [18], neuroanatomical [19–21], prescription rate [2, 22], and emotional challenges [4] in female compared to male ADHD. Girls and women with ADHD often face unique comorbidities: cognitive [23], binge eating [24], chronic fatigue [7], social [25], substance use [7], anxiety, and mood comorbidities [4, 7, 8, 15, 26, 27]. The management of ADHD in females may be suboptimal, where underdiagnosis and referral bias predominate [28], and the following areas are neglected: differences in symptom presentation [7], research prioritising sex differences in ADHD [29], and finally, the implications of gender differences in the psychosocial treatments of ADHD [30]. Failure to recognise and diagnose ADHD in women can result in prolonged periods of impaired self-esteem, difficulties in interpersonal relationships, challenges with self-regulation, and increased self-blame [4].

## ADHD and female sex: A theoretical framework of hormonal interplay

Biologically, an underlying theoretical framework suggests that varying oestrogen levels modulate the dopaminergic neurotransmission that is involved in ADHD pathophysiology [31]. This hormonal influence extends beyond ADHD, with the (pre)menstrual phase consistently associated with symptom exacerbation across various psychiatric conditions, including psychosis, depression, suicidality, and alcohol use disorders [32].

In ADHD, periods of lower circulating oestrogen are believed to impact dopaminergic neurotransmission negatively, leading to cyclical variations in symptom severity [4, 7] and diminished premenstrual treatment response to psychostimulants [33]. ADHD symptoms in women may exhibit fluctuations corresponding to hormonal changes during the menstrual cycle, with potential exacerbation during the premenstrual phase. Consequently, the timing of diagnostic assessments and treatment response evaluations in relation to the menstrual cycle may significantly influence both the establishment of an ADHD diagnosis and the perceived efficacy of interventions. This temporal relationship underscores the importance of considering hormonal fluctuations when assessing and treating ADHD in adult women [28]. We propose that researchers and clinicians systematically consider the menstrual cycle phase and hormonal status to capture accurately the dynamic nature of ADHD symptoms in women and optimise therapeutic outcomes.

Since 2002, our specialised adult ADHD clinic treats approximately 1000 adults with ADHD per year, of which about 60% are women. Our team includes psychiatrists, clinical psychologists, doctors, and nurse practitioners, who have extensive experience in the assessment and treatment of ADHD in women across the lifespan, including expertise in hormonal and reproductive mental health. Some of our female patients report monthly fluctuating symptoms. We recently published a case series describing a small group of women with premenstrual worsening of ADHD and mood symptoms as well as less effect of current ADHD medication premenstrually [32]. Additionally, we treat many female patients who are diagnosed for the first time with ADHD as they present with (peri)menopausal exacerbation of symptoms. Our clinical experience has informed several research initiatives, including a pilot questionnaire distributed at a 2016 conference for women with ADHD (*n* = 200), revealing a two- to three-fold increase in premenstrual, postpartum, and (peri)menopausal mood symptoms compared to the general Dutch population (unpublished data).This was confirmed by our subsequent study in women with a formal diagnosis of ADHD [31].

In this study, we offer a clarifying framework to understand fluctuating symptom levels in female ADHD as well as practical tools for research and clinical practice.

## Methods

### Clinical expertise and protocol development process

This protocol was developed collaboratively by the three authors in an iterative manner: DW and SJJSK are psychiatrists with a background in adult ADHD research, including biological rhythms and female-specific aspects of ADHD. SJJSK is a professor of adult ADHD and was a co-founder of the Head Heart Hormones (H3)-Network in the Netherlands, an interdisciplinary collaboration aimed at improving women’s health care, amongst General Practitioner (GPs), psychiatrists, gynaecologists, and cardiologists treating women. In addition, DW and SJJSK are editor and founder (respectively) of the DIVA Foundation, providing a semistructured clinical interview for diagnosing ADHD translated into more than 30 languages. MdJ is a medical doctor, cultural analyst, and doctoral researcher currently analysing data from a large online survey of women with ADHD.

Clinical insights were informed by direct patient care (including many women presenting with (peri)menopausal exacerbation of symptoms and first-time ADHD diagnoses in adulthood), regular multidisciplinary case discussions, and ongoing participation in national and international ADHD research networks.

### Literature review and integration

To complement clinical experience, we conducted a targeted literature review focusing on hormonal influences on (1) ADHD symptomatology in women and (2) diagnostic and treatment considerations. Relevant references were identified through PubMed and Embase searches (keywords: “ADHD,” “women,” “hormones,” “menstrual cycle,” “menopause,” “diagnosis,” “treatment”), as well as through the review of bibliographies from key articles and clinical guidelines published between 2000 and April 2025. Key findings from the literature were synthesised and integrated with clinical observations to inform the development of practical tools and recommendations. Where appropriate, we also consulted online sources identifying similar research gaps in women with ADHD [29].

### Protocol development process

Initial protocol drafts were based on observed clinical patterns and unmet needs, particularly regarding symptom fluctuations across hormonal transitions. These drafts were refined through discussion and updated to reflect emerging evidence from the literature.

## Results

### Review: Periodical worsening of symptoms

We identified four articles meeting our search criteria in PubMed and Embase (published 2000–2024) that directly addressed hormonal influences on ADHD symptomatology, diagnosis, or treatment in women [30, 34–36].

A systematic review described the relationship between sex hormones, reproductive stages, and ADHD. It concluded that hormonal transitions (puberty, menstruation, pregnancy, and menopause) can significantly influence symptom severity and treatment response, but that empirical data remain sparse [30].

### Review: Menstrual cycle and symptom fluctuation

A qualitative study exploring the lived experiences of women with ADHD regarding the menstrual cycle’s impact on their symptoms [34]. Participants consistently reported that ADHD symptoms – particularly inattention, emotional dysregulation, and executive dysfunction – worsened during the late luteal and menstrual phases, coinciding with declining oestrogen levels. Many women perceived reduction in the efficacy of their usual ADHD medication during these phases. Two other studies described how cognitive functions and ADHD symptoms may fluctuate with hormonal changes throughout the menstrual cycle [35, 36]. The first highlighted that ADHD symptoms were highest during the early follicular and early luteal (postovulatory) phases, when oestrogen levels are low or rapidly declining, especially in women with high trait impulsivity [35]. The second proposed that the interaction between oestrogen and the neurotransmitters dopamine and noradrenaline, could underlie some clinical features of ADHD in women [36]. Oestrogen modulates these neurotransmitter systems and may influence cognitive and emotional regulation [37].

### Pharmacotherapy adjustments

We previously published a community case series, where nine women with ADHD and premenstrual symptom worsening underwent individualised increases in psychostimulant dosage during the premenstrual phase. All participants experienced improvements in ADHD and mood symptoms, with minimal adverse events. Premenstrual inattention, irritability, and energy levels improved to resemble those of non-premenstrual weeks, and all women opted to continue with the adjusted regimen [33].

### Clinical protocol

#### Assessment

Based on our clinical experience, we recommend menstrual cycle awareness: consider menstrual phase when assessing symptoms and treatment response. At baseline evaluation, routine assessment should include the first day of last menstruation, average cycle length, and use of hormonal contraception or hormone therapy. The current menstrual phase (follicular or luteal) should be determined to contextualise symptom severity and treatment response. Daily tracking should continue throughout treatment to monitor symptom fluctuations across the cycle. ADHD and mood symptoms should be tracked daily for at least 2 months. A premenstrual syndrome (PMS) calendar and a scale for the severity of premenstrual dysphoric disorder (PMDD) symptoms are helpful here (see Table 1). Useful menstrual cycle applications include Clue, Euki, Flo, Glo, Natural cycles, and Periodical. Comorbidities such as PMDD, (peri)menopausal depression, anxiety, somatic symptoms, and sleep disorders should be screened for, using validated questionnaires. The MINI-Plus has been validated across diverse populations and settings, demonstrating its diagnostic accuracy for a range of psychiatric disorders, including PMDD. It is particularly effective in clinical environments where comprehensive psychiatric evaluation is necessary and is considered the gold standard [45]. During the menstrual cycle and female lifespan, fluctuating ADHD symptom frequency and severity should be assessed. The ADHD Rating Scale (ADHD-RS) is widely used and determines the impact on life activities [46]. For monitoring of comorbid symptoms, questionnaires such as the Quick Inventory of Depressive Symptomatology (QIDS, for depressive symptom severity) have been validated in adults and is sensitive to treatment changes [47]. Rating sleep quality from 0 to 10 can be useful to track fluctuating symptoms of sleep problems. Postpartum, females with ADHD should be screened for depression using the Edinburgh Postnatal Depression Scale, which has been validated in various adult populations, including community samples [48]. Women with ADHD who are above 40 years should also be screened for (peri)menopausal symptoms, using the validated Greene Climacteric Scale, [49]. Furthermore, the Menopausal Rating Scale also effectively measures menopausal symptoms and is valid in comparison to established tools [50] (Table 1).

**Table 1.** Assessment tools and treatment options for ADHD and comorbidities according to the female life phases: The menstrual cycle, postpartum, and (peri)menopause

Timing	Action	Outcome	Treatment options
Before intake	Complete PMS calendar for 2 months	Diagnose comorbid PMDD (symptoms)	
Baseline evaluation	Note the first day of last menstruation	Determine current menstrual phase	
	Relate to cycle day at time of assessment	Evaluate current endorsement of symptoms in context of current menstrual cycle phase	
	MINI-Plus [38]	Diagnose comorbid psychiatric disorders PMDD Depression Anxiety Sleep disorders Eating disorders, etc. Note: Premenstrual exacerbation of symptoms of all disorders	
Evaluation during ongoing treatment: premenopause	Complete PMS calendar or phone application	Monitor fluctuating ADHD and mood symptoms in relation to cycle phase to evaluate PMDD	Combined oral contraceptive pill without stop week Increased stimulant dosage in luteal phase Selective Serotonin Reuptake Inhibitor (SSRI) (cyclical or continuous, preferred)
	Visual Analogue Scale	Measure intensity of premenstrual symptoms	
	ADHD-RS [39]	Rate fluctuating ADHD severity	
	QIDS [40]	Determine depressive symptom severity	
	Edinburgh Postnatal Depression Scale [41]	Rate postpartum mood symptoms	Psychotherapy; SSRI
(Peri)menopause [41]	Greene Climacteric Scale [42] Menopause Rating Scale [43]	Rate (peri)menopausal symptoms	
	Determine need for MHT	Care pathway, EMAS [44]	Psychoeducation MHT Antidepressant
	Rate sleep quality 1–10	Monitor sleep premenstrually, postpartum, and (peri)menopausally	Sleep hygiene Treat comorbid sleep disorders

EMAS, European Menopause and Andropause Society; MHT, menopausal hormone replacement therapy; PMS, premenstrual syndrome; PMDD, premenstrual dysphoric disorder.

#### Treatment considerations

During treatment, we recommend psychoeducation about the impact of menstrual cycle and hormonal transitions on ADHD symptoms, if possible, in a group setting [51]. Ongoing cycle and symptom tracking may foster better insight and self-management. We suggest monitoring ADHD medication effectiveness across the menstrual cycle and considering increasing psychostimulant dosage in the luteal phase if premenstrual worsening of ADHD and mood symptoms occur, with individualised dosing and careful monitoring. Increased psychostimulant dosage is not a substitute for SSRIs in depressive symptoms, or oral contraceptives for somatic complaints, but may be used complementarily. For comorbid PMDD, we recommend considering SSRIs (either luteal phase only or continuously) and psychotherapy. Combined oral contraceptives (without a stop week) may be considered for somatic symptoms and to stabilise hormone levels. For women over 40 years or with (peri)menopausal symptoms, hormone therapy and/or ADHD medication for new or worsening cognitive and mood symptoms can be considered. Cognitive Behavioural Therapy (CBT) and antidepressants (SSRIs, Selective Serotonin-Noradrenaline Reuptake Inhibitor (SNRIs), bupropion) may be considered for mood and somatic symptoms (Table 1).

## Discussion

Women with ADHD appear to have periodical worsening of symptoms, closely related to hormonal fluctuations [28, 31, 33]. Therefore, female-specific research and clinical practice require different approaches as women transition between different life phases. Adequate treatment of females with ADHD requires consideration of the impact of hormonal fluctuations [1, 7].

With a targeted literature review, we examined hormonal influences on ADHD symptomatology, diagnosis, and treatment in women. We identified four primary studies directly addressing these influences [30, 34–36]. The literature is therefore sparse and much remains to be clarified in this field.

The qualitative study we reviewed suggested that ADHD symptoms worsened during the late luteal and menstrual phases, coinciding with declining oestrogen levels [34]. Our own patients perceived reduction in the efficacy of their usual ADHD medication during these phases [33]. As described in two other studies reviewed, cyclical hormonal fluctuations, and especially rapid declines in oestrogen, appear to exacerbate ADHD symptoms [35, 36]. Fluctuations in oestrogen and progesterone have been shown to affect ADHD symptom severity and executive functioning significantly across the menstrual cycle [8, 35]. Oestrogen may modulate neurotransmitter systems central to attention and executive function. Through its effects on dopamine pathways, it is also reported to impact inhibitory control, which is relevant to emotional regulation [36]. In adulthood, girls and women with ADHD experience more emotional dysregulation than males with ADHD. Emotional dysregulation in ADHD is often misattributed to other disorders, such as (bipolar) depression or personality disorders [8]. Women and girls frequently internalise their symptoms, which may result in comorbid depression, anxiety, and eating disorders [4, 7–9, 27]. One study found that girls with ADHD are 2.5 times more likely to be diagnosed with major depression than their female peers without ADHD [52]. It is plausible that the additional impact of hormonal fluctuations on the female body and brain account for (some of) the observed sex differences. Combined, these findings support the hypothesis that cyclical hormonal fluctuations, especially in oestrogen, can exacerbate ADHD symptoms in women and influence treatment response.

### Assessment

To provide a more comprehensive perspective and contextualise our findings, we also make recommendations for assessment and treatment based on our clinical experience and other key articles related to this topic. This broader approach enables us to address several additional themes relevant to female-specific ADHD research and clinical practice.

When evaluating ADHD for the first time, clinicians and researchers should routinely ask about the first day of the last menstruation and cycle length, or use of hormones [53]. With this information in mind, it can be determined whether the woman is in the follicular or luteal phase of her menstrual cycle. The relationship between the current endorsement of symptoms and the current menstrual cycle phase may give insights into why the symptoms are particularly severe at a specific time: typically, ADHD and mood symptoms are most intense around ovulation and in the late luteal phase. They diminish after the first few days of menstruation [31, 33]. Also, the timing of the evaluation with regard to the menstrual cycle phase may shed light on current treatment response and guide dosing of psychostimulant medication.

As described earlier, ADHD commonly co-exists with symptoms of PMDD [31]. All women with ADHD should be screened for PMDD, and to identify a possible diagnosis, women should complete a PMS calendar for at least 2 months. By tracking ADHD and mood symptoms in relation to their menstrual cycle, they may gain insights into the impact of the various menstrual phases on fluctuating symptom severity of mood symptoms. We routinely include this in the psychotherapy group we have designed for with ADHD and PMDD [51]. During their ongoing treatment and in research studies, women with ADHD should continue to use the PMS calendar or smart phone applications for reporting cycle phase [51]. These tools will improve the reliability and validity of clinical and research findings. Monitoring symptom fluctuations per menstrual cycle phase may also be relevant to other (co-existing) psychiatric disorders, such as autism and bipolar disorder [1, 32].

Female-specific norms may need to be developed for the different phases of the menstrual cycle to prevent what we have noted from clinical experience: underdiagnosis of ADHD in the follicular phase of the cycle and undertreatment in the luteal phase. We recently published an article with several colleagues using this as a starting point [54].

While the average age for menopause is 51 years, a genome-wide association study showed that women with ADHD may have an earlier menopause [55], making them vulnerable to the vasomotor (thermoregulatory problems such as hot flushes and night sweats), somatic (palpitations, fatigue, joint pain, insomnia), psychological (depressed or anxious mood, irritability), and genitourinary symptoms (vaginal dryness, dyspareunia, urinary frequency, urgency, or incontinence). Specific attention should be paid to sleep in this population, as women and men with ADHD have chronic sleep problems, which often worsen in women during (peri)menopause [56]. Disrupted sleep can worsen concentration and mood problems. Once (peri)menopausal symptoms occur, care should be taken to distinguish new-onset “brain fog” and cognitive problems from an exacerbation of previously undiagnosed ADHD but present from childhood.

### Monitoring and treatment

The two pillars of gold standard ADHD treatment are psychological and pharmacological interventions. Both warrant a female-specific approach [1].

The menstrual cycle in women (with ADHD) needs to be discussed openly, breaching taboo. Premenstrual depressive symptoms worsen self-esteem and clinical outcomes time after time, if they remain unaddressed [4]. In addition, for women who lack a sense of timing and an overview, gaining insights into the fluctuation of ADHD symptoms is a particularly valuable first step. Simultaneously, the effectiveness of ADHD medications should be monitored across the cycle and, if necessary, adjusted [33]. We base this suggestion on our case study; however, a randomised controlled trial is necessary to have more certainty on the efficacy of premenstrual psychostimulant dose adjustment. When there is a comorbid diagnosis of PMDD, our suggestion of an SSRI (in the luteal phase only or preferably continuously) is supported by the literature [57]. Other studies suggest a combined oral contraceptive, particularly where somatic symptoms predominate [58]. We favour oral contraceptive use, without a stop week, to stabilise hormone levels, based on our clinical experience. However, this has not been supported by the literature, and more research is needed [59].

For monitoring of symptoms during the menstrual cycle, questionnaires as cited in Table 1 are useful for ADHD, PMDD, depressive symptom severity, and rating sleep quality to track fluctuating symptoms. Systematic monitoring is important because it enables identification of cyclical patterns in symptom exacerbation, thereby supporting more precise diagnosis and facilitating individualised treatment adjustments, such as optimising medication timing or dosage. This approach also encourages patients to recognise and understand their own symptom fluctuations, enhancing engagement and communication with clinicians.

In the case of peri/postnatal depression, in addition to support and psychoeducation, SSRIs, certain stimulants, or other therapies can be used [60, 61], but full discussion of these is beyond the scope of this report.

For women who have compensated for their ADHD symptoms since childhood, sleep and executive function difficulties arising in the (peri)menopause may unmask an underlying ADHD diagnosis, in which case a combination of hormone therapy and ADHD medication is warranted. In addition, for (peri)menopausal exacerbation of ADHD, low mood, sleep, and somatic symptoms [7, 8, 30, 31], CBT [62], and antidepressants such as SSRIs, SNRIs [63], and bupropion can be considered. Also, mood, vasomotor and somatic symptoms, sleep disturbances, and sexual dysfunction during the (peri)menopause may improve with menopausal hormone replacement therapy [64]; however, this has not been specifically studied in women with ADHD. Hormone replacement is not currently approved for the treatment of (peri)menopausal depression, because of insufficient evidence, although a recent retrospective study of peri- and postmenopausal women suggested significant improvement of mood using Hormone Replacement Therapy (HRT) [65], but placebo-controlled studies are necessary. The “new” onset of executive function difficulties in the (peri)menopause should be investigated for underlying, undiagnosed ADHD.

### Reflections on protocol development and implementation

Developing this female-specific ADHD protocol highlighted several challenges, particularly the limited availability of research or validated tools sensitive to hormonal fluctuations and the need for greater awareness among clinicians regarding the impact of the menstrual cycle on ADHD symptoms. Ensuring consistent engagement with symptom and cycle tracking can also be difficult for women with ADHD, who may already struggle with organisation and motivation.

Despite these challenges, implementing such a protocol offers clear benefits. It enables more accurate assessment and tailored treatment by accounting for cyclical symptom changes. It empowers women to understand and manage their condition better, possibly even improving treatment adherence. However, practical barriers such as the need for additional clinician training and the variability of menstrual cycles, especially in younger women, must be considered.

From a developmental perspective, while this protocol is primarily designed for adult women, its principles can be adapted for use in adolescent girls, particularly from the onset of menarche. Early incorporation of menstrual cycle tracking and symptom monitoring could facilitate earlier identification of ADHD and its comorbidities in girls, who are often underdiagnosed. Future research should focus on validating these approaches in younger populations and exploring how hormonal context can inform early intervention strategies, ultimately improving outcomes for females across the lifespan.

## Conclusion

The theoretical context presented here serves as a starting foundation for addressing the unique challenges faced by women with ADHD in clinical practice and research. Recognising the impact of the female hormones on the symptom severity of ADHD, mood, and potentially other disorders, is essential for advancing our understanding of ADHD in women. Future research should prioritise including the menstrual cycle phase in investigations and clinical treatment, screening for premenstrual and postpartum depression in women with ADHD, as well as screening for early-onset (age <45 years) and severe (peri)menopausal symptoms, to develop more effective treatment strategies for women with ADHD. Additionally, efforts to adapt and validate these approaches for girls and younger females could facilitate earlier identification and intervention, leading to better care for women and girls with ADHD across the lifespan. Finally, while this study focuses predominantly on the impact of biological sex, the impact of gender roles and gendered psychosocial expectations should not be neglected.
